# Exploring the Role of In-Person Components for Online Health Behavior Change Interventions: Can a Digital Person-to-Person Component Suffice?

**DOI:** 10.2196/jmir.8480

**Published:** 2018-04-11

**Authors:** Sara Santarossa, Deborah Kane, Charlene Y Senn, Sarah J Woodruff

**Affiliations:** ^1^ Department of Kinesiology University of Windsor Windsor, ON Canada; ^2^ Faculty of Nursing University of Windsor Windsor, ON Canada; ^3^ Department of Psychology University of Windsor Windsor, ON Canada; ^4^ Department of Women’s and Gender Studies University of Windsor Windsor, ON Canada

**Keywords:** digital person-to-person, in-person, online intervention, behavior change, health, digital media, health care

## Abstract

The growth of the digital environment provides tremendous opportunities to revolutionize health behavior change efforts. This paper explores the use of Web-based, mobile, and social media health behavior change interventions and determines whether there is a need for a face-to-face or an in-person component. It is further argued that that although in-person components can be beneficial for online interventions, a digital person-to-person component can foster similar results while dealing with challenges faced by traditional intervention approaches. Using a digital person-to-person component is rooted in social and behavioral theories such as the theory of reasoned action, and the social cognitive theory, and further justified by the human support constructs of the model of supportive accountability. Overall, face-to-face and online behavior change interventions have their respective advantages and disadvantages and functions, yet both serve important roles. It appears that it is in fact human support that is the most important component in the effectiveness and adherence of both face-to-face and online behavior change interventions, and thoughtfully introducing a digital person-to-person component, to replace face-to-face interactions, can provide the needed human support while diminishing the barriers of in-person meetings. The digital person-to-person component must create accountability, generate opportunities for tailored feedback, and create social support to successfully create health behavior change. As the popularity of the online world grows, and the interest in using the digital environment for health behavior change interventions continues to be embraced, further research into not only the use of online interventions, but the use of a digital person-to-person component, must be explored.

## Introduction

### Background

Several aspects of the digital environment offer opportunity to support behavior change efforts, including reach, engagement, accessibility, collaboration and advocacy, and research potential [1]. Notably, there has been an increased interest from both public health organizations and those in academia, around using Web-based, mobile, and social media health behavior change interventions. It is believed that these popular digital media channels can play a valuable role in leveraging health messaging and consequently, behavior change. Although traditional face-to-face interventions or interventions with in-person components are (and continue to be) successful in health behavior change [2,3], traditional approaches can present with various barriers such as logistic problems, a challenge of keeping participants actively engaged, can be labor intensive, and expensive to scale for larger populations. Using components of the digital environment may offer solutions to traditional challenges because of their low cost, high reach, anonymity, adaptability, and scalability [4]. Furthermore, comparisons of online interventions with traditional face-to-face interventions indicate that online treatment is generally at least as effective as conventional approaches and also possess several advantages [5-7]. Similarly, supplementary literature within the health behavior change domain has shown no significant treatment differences between the face-to-face and the online intervention groups [5-8], suggesting that online-only interventions may be just as valuable as face-to-face interventions. However, as a majority of online interventions are used in adjunct to traditional approaches [1], there is a need to understand what role in-person components play in online interventions. Furthermore, research indicates that the effectiveness of, and adherence to, online interventions is enhanced by human support [9-11]. As intervention adherence is important in predicting behavior change, the inclusion of a digital person-to-person component for an online behavior change intervention can help to combine the effectiveness and socialization opportunities of in-person meetings with the technologically enhanced active learning possibilities of the digital environment [1,5-8].

**Figure 1:**
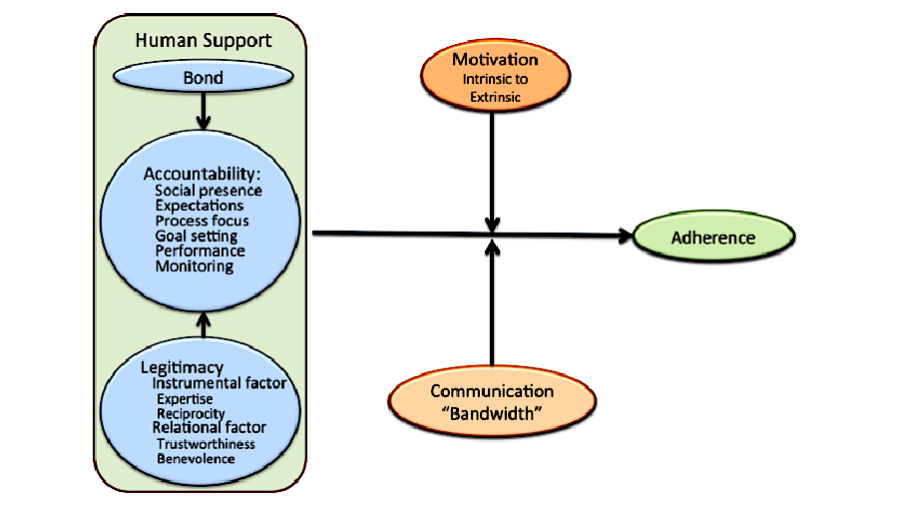
Model of supportive accountability (Mohr, Cuijpers & Lehman [11]).

Perhaps, the dynamic, socially supportive, and interactive elements of digital media channels (ie, Web, mobile, and social media) may obviate the need for further interpersonal in-person components [6], as a digital person-to-person component can be used to cultivate a similar interpersonal connection, while overcoming the barriers of face-to-face interventions. Online human-supported interventions or digital person-to-person components have, in recent meta-analyses [12,13], obtained larger effect sizes than online self-guided programs, suggesting a need to further explore the role of the digital person-to-person relationship. For the purpose of this viewpoint paper, a “digital person-to-person” component will encompass any type of online feature that creates a sense of interpersonal connection or virtual interaction, thus, embodying qualities of a physical in-person or face-to-face components such as guidance, feedback, and support. For example, a digital person-to-person component can include human support provided through peers via online message groups or by posting or reading bulletins [14], as well as other elements of online systems that create social support such as chat forums and/or chat rooms [15]. Similarly, the digital person-to-person component can be offered on a one-to-one basis through virtual coaches, therapists, counselors, or facilitators via email, instant messaging sessions (eg, text message), and teleconferencing (eg, webcam and Skype). Digital person-to-person features create accountability, feedback, and social support, emulating traditional, physical in-person or face-to-face components and can foster motivation, encouragement, and commonality [16]. Furthermore, expectations about the digital person-to-person, such as accountability, feedback, and social support, may be grounded in social or behavioral theories including the theory of reasoned action [17] and the social cognitive theory [18], while being guided by the human support constructs of the model of supportive accountability (Figure 1) [11].

### Objectives

Thus, the purpose of this viewpoint paper is to suggest that based on a comprehensive review of the literature, there is a need for a face-to-face component in Web-based, mobile, and social media health behavior change interventions but that a digital person-to-person component can foster similar results while dealing with challenges faced by traditional intervention approaches.

## Methods

To delineate the scope of the study and make it more replicable, the focus was on published references searchable through major bibliographic databases. This review adhered to the defining characteristics of Web-based, mobile, and social media health behavior change interventions, and this review excluded unpublished and untested programs. Due to the emerging state of Web 2.0 research, this viewpoint paper will not limit studies further by methodology, being inclusive of study design, participant, and setting. In particular, a wide variety of platforms were included in the search, including blogs and microblogging technologies, social networking sites, video sharing programs, and mobile health (mHealth) apps. Literature search strategies were developed using subject headings related to Web-based, mobile, and social media health behavior change interventions. Proquest Social Sciences, Web of Science, PsycINFO, Scopus, and PubMed were searched for “digital person-to-person,” “in-person,” “online intervention,” “behavior change,” “digital media,” “health care,” “social media,” and “Web 2.0” from 2004 to April 2017. The search began with studies published since 2004 because that is when the term “Web 2.0” was coined to describe the shift to a more participatory online landscape. However, studies before 2004 were used to develop an understanding of the impact of in-person and/or traditional therapies or interventions. Finally, to ensure literature saturation, the reference lists of included studies or relevant reviews identified through the search were scanned.

## Results

### Using Digital Media Channels in Health Behavior Change Interventions

The digital environment consists of digital media, and although difficult to define, partly because it is ever changing, digital media in its broadest sense are content that can be transmitted over the internet or computer or phone networks [1]. Digital media channels such as the internet, mobile phones, and social media have become increasingly popular and have altered the nature of interactions around health issues. A once-passive one-way transfer of information, now it has become a network of multidirectional conversations [19]. The sense of interaction and multidirectional communication [20,21] offered by digital media channels cultivates active engagement [22-24] and information dissemination to a larger number of individuals [25]. On the basis of the popularity of these channels, an opportunity is presented to connect with individuals in their daily lives on issues concerning health and health behavior change [1].

Vast numbers of North Americans use the internet daily [26,27]. As presented in a meta-analysis of 5 papers, the literature presents substantial evidence that the use of Web-based interventions improves behavior change outcomes [28]. Web-based health interventions can be defined as “primarily self-guided intervention programs, delivered through a website, aiming to create positive change and/or improve or enhance knowledge, awareness, and understanding” [14]. Specific behavior change techniques of Web-based interventions may include real-time support, goal setting tools, alarms, reminders, and platforms to share with friends or family [1]. Particularly with health behavior change, Web-based interventions have seen several successful outcomes, including increased exercise time, increased knowledge of nutritional status, increased knowledge of asthma treatment, increased participation in health care, slower health decline, improved body shape perception, weight loss maintenance [28], and weight loss [6,29]. It has been proposed that structured (ie, lessons and activities) Web-based interventions are able to replicate health outcomes expected of a traditional, in-person intervention [30] and tend to be a more cost-effective approach [31]. However, other literature has shown that although Web-based interventions resulted in greater behavior change compared with control conditions (ie, waitlist or usual care), they had significantly less change compared with face-to-face interventions [32-34].

Particularly in North America [26,35], mobile phones are becoming a primary means of online access, as vast majority of individuals now own a mobile phone. Mobile phone are recommended as a good access point for a health behavior change intervention, since usage is high across various populations, including those considered to be underserved (ie, racial or ethnic minorities, youth, low and social economic status). [1,19,20,36]. mHealth interventions involve the use of mobile computing and communication technologies such as mobile phones, personal digital assistants, tablets, and portable media players to disseminate health information [37]. Subsequently, mHealth interventions are successful in creating health behavior change, as well as higher patient adherence, satisfaction, and acceptability than Web- or paper-based interventions [38]. Specifically, mHealth interventions have shown small but positive effects on weight loss behavior [39] and are a promising tool for decreasing risky sexual behaviors and drug use [40]. Furthermore, the use of tailored text messages as an adjunct to an in-person multidisciplinary weight management intervention resulted in improved feasibility, acceptance, and adherence [41]. The use of mobile phones offers health professionals an opportunity to engage with patients and colleagues on a scale when and where people are open to communicating and perhaps behavior change [42]. Future research on the effectiveness of text message delivery characteristics is needed to establish longer term intervention effects [43]. Moreover, the acceptance of mobile phones has helped to increase the popularity of online interactive platforms such as social media [44].

Social media are a broader concept that encompasses sites that allow users to generate and share content [21]. There are 6 main social media platforms, which include blogs, social networking sites, virtual worlds, online collaborative projects, content communities, and virtual game worlds [45]. As the use of social media continues to rise [26,46], it may indicate the potential for its role as a tool in the public health care system, specifically, health behavior change interventions. Social media platforms have been found to be successful in health behavior change interventions, with meta-analyses finding that the direction of effect for the primary outcomes favors interventions with social media components [45] and a slight positive effect of social networking site interventions on health behavior change outcomes [47]. However, there is a lack of clear evidence of the effectiveness of social media in behavior change interventions [24,47,48], as most studies are not measuring an isolated effect of social media, thus creating a lack of ecological validity [49]. Furthermore, challenges of social media can include the spreading of misinformation and privacy breaches [45], which might suggest that using social media alone may be insufficient to promote health [48].

### Role of In-Person Components in Online Health Behavior Change Interventions

It has been suggested that using a face-to-face approach is the “gold standard” in behavior change interventions [50]. Face-to-face interactions have greater bandwidth (ie, the number of communication cues a medium can convey), and this can lead to a greater ability to complete tasks, better interpersonal relations, and greater social presence [14]. Combining the verbal, nonverbal, and contextual cues of face-to-face communication could be assumed to provide the richest source of information and perhaps most positively influence behavior change. Furthermore, in face-to-face interventions, the human support created by the in-person component offers the core of the intervention while simultaneously coordinating a relationship with the participant in a way that will efficiently promote the use of the interpersonal connection to continue in the intervention [11]. In contrast, online behavior change interventions separate the content of the treatment, which is provided in a standardized manner via a website, mobile device, or social media platform, from support provided by humans, which is often intended to increase adherence [51-53]. However, Web-based, mobile, and social media interventions have shown promising results in health behavior change, but a majority of the literature focuses on these digital media platforms used in adjunct to traditional approaches [1]. The combination of online and face-to-face interventions may be reflective of the argument that it should not be necessary for online interventions to prove more effective than face-to-face treatments but rather to provide close to equivalent benefits and outcome results [50], thus, implying that online interventions are meant to provide an alternative or adjunctive component to already well-established and highly effective face-to-face interventions.

As such, some literature suggests that the idea of having a combination of online and face-to-face components within an intervention or program is ideal [1,6,54]. For example, a weight loss study that used Facebook to provide social support between monthly in-person meetings found that engagement in the Facebook support groups was significantly associated with weight loss during the 4-month maintenance period of this study, even after adjusting for face-to-face meeting attendance [55]. Furthermore, in a meta-analysis that focused on Web-based interventions and weight loss, it was reported that additional weight loss occurred when Web-based interventions were used to supplement face-to-face interventions; however, substituting face-to-face interventions with Web-based interventions resulted in significantly less weight loss [56]. These findings suggest that when digital media channels are used in conjunction with traditional approaches such as in-person behavior change interventions, they tend to be beneficial components [57] and perhaps will not be as successful if used alone. Conversely, it is important to note that some reviews have concluded that a meta-analysis could not reliably detect the effectiveness of online interventions because of the heterogeneity of designs and the small number of comparable studies [58-60].

Only a few Web-based, mobile, and social media interventions have truly measured behavior change; overall, there is a lack of comprehensive evaluation [1]. In the limited studies that investigated solely online interventions, it was recommended that having an in-person component could increase engagement and allow participants to interact and get to know each other before expecting them to interact online [61]. In addition, online behavior change interventions have had higher fidelity (ie, actual usage or intended usage of the online component) when participants knew each other before recruitment [24]. Specifically to social media, considering an in-person component has been suggested because of the “stranger phenomenon” [49]. This is based on the idea that social media are currently being used for conversations and maintenance of existing relationships and thus not being used to cultivate new acquaintances (ie, strangers). Supplementing with a face-to-face meet up (ie, more traditional way of forming acquaintances) may help overcome this particular barrier of social media. In a small pilot intervention study utilizing Facebook, which included an optional face-to-face meeting of all participants, only 3 of 8 participants attended [16]. Moreover, similar rates of participation existed in those who did not attend the in-person meeting compared with those who did [16]. Thus, further research into the need and role of in-person meetings in online interventions is warranted.

Albeit, many online interventions have supplemented with some form of in-person meeting or counseling [4,6,62]. These face-to-face interactions can be time-consuming, inconvenient, and logistically challenging. Research suggests that the use of a virtual health coach or online communication with a counselor or facilitator can be just as effective in behavior change as an in-person interaction [63,64], with implications for cost savings. Similarly, online interventions offer a promising alternative to traditional peer interventions, home visits, and/or pediatric office-based strategies to promote healthful behaviors [16], as online participants can interact frequently and at their convenience, a pattern that facilitates engagement, retention, and delivers a high intervention dose at a low cost with minimal resources [20]. These digital media channels provide a mechanism for participants to receive new information instantaneously, obtain immediate personalized automated feedback, and interact within a virtual group network, while at the same time allow for flexibility around work or school schedules and childcare responsibilities [65]. In addition, the potential anonymity of an online intervention group and its faceless quality allows participants to feel valued for the strength of their contributions rather than being evaluated on their physical appearance or disabilities [66]. Participants are likely to feel empowered, and in a safe environment, where they are able to digest the information at their own pace and better use it to enhance behavior change efficacy. However, the effectiveness of and adherence to online interventions is enhanced by human support [9,10]; and thus, the positive findings of online interventions coupled with the drawbacks of in-person components present an opportunity for the digital person-to-person.

### Using a Digital Person-to-Person Component

For a digital person-to-person component to be considered and be successful, certain adjustments need to be examined. During in-person meetings, participants are able to view nonverbal communication cues, including body language and voice qualities. These nonverbal cues may not be as obvious in online interactions. Several steps can be taken to overcome this limitation and to maintain accurate and a more complete understanding between the participant and the digital person-to-person component. Strategies to overcome nonverbal cues absent in a digital person-to-person component may include extended wording, various stylistic procedures for emphasizing text and using emoticons [14]. Extending wording and verbal expressions can help clarify messages, and the use of emoticons can enrich messages by mimicking a missing intonation or gesture [14,67,68]. In addition, participants should be well aware of the fact that messages may be misunderstood, hence a need for more probing and clarifications than in face-to-face sessions [14]. Furthermore, although possible and effective in online communications, the expression of feelings is not as automatic as in in-person meetings or relationships. This means that the digital person-to-person component must consciously consider using words and expressions that might not be used in face-to-face contact, to communicate empathy, care, concern, and warmth toward participants [14]. Again, participants have to be aware that their feelings are not as obvious and vivid as they would be in a face-to-face meeting [14]. Overall, the possibility does exist that if thoughtfully executed, a digital person-to-person component can perhaps be leveraged to substitute in-person and face-to-face components of online behavior change interventions, while overcoming traditional barriers and maintaining a sense of interpersonal connectedness. Moreover, the possibilities about the digital person-to-person relationship and opportunities for a successful alternative to in-person meetings are grounded in theory.

Research has not only shown a positive effect of grounding behavior change interventions in theory [48], but it is suggested as a necessity [1,62]. Using a digital person-to-person component in the delivery of an online behavior change intervention allows one to incorporate the best features of in-person interaction and the live instruction to personalize learning, allow thoughtful reflection, and differentiate instruction from participant to participant across a diverse group of learners. Thus, using a digital person-to-person component may be grounded in social and behavioral theories such as the theory of reasoned action [17] and the social cognitive theory [18], while being guided by the human support constructs of the model of supportive accountability (Figure 1) [11].

The theory of reasoned action [17] predicts that norms of significant people in an individual’s social circles (ie, subjective norms) have a strong impact on the influence in the individual’s behavioral intentions. In the digital media literature, descriptive norms, which are similar to engaging in social comparison (ie, comparing if you should or should not engage in a behavior based on what others like you are doing), are found to be more powerful in behavior change than injunctive norms [48,62]. Moreover, digital media channels such as social media thrive off social comparison and can motivate user participation by a desire to belong [69,70]. Similarly, the social cognitive theory [18] predicts social learning by observation, which can take place in both online and offline social networks. For example, the use of virtual coaches or facilitators can provide positive reinforcement for participation and model desirable behavior outcomes (ie, photographs, videos) [16]. The social cognitive perspective of social support proposes that perceived support (ie, an individual’s belief that he or she is well supported) leads to better coping skills and higher self-esteem [71]. In addition, the social cognitive theory encompasses the idea of social diffusion and innovation as the way new ideas or cultural practices are transmitted or reinforced throughout a society [18]. According to Bandura [18], social innovation and diffusion can only be reinforced by media (eg, digital media) but not innovated; it is influential people who create innovation. Bandura [18] also suggested that the more media dominate people’s lives, the more they will learn from it and less from people. Hence, there is a need for a digital person-to-person element in online behavior change interventions. Finally, using the human support constructs of the model of supportive accountability [11], it is suggested that the role of the digital person-to-person component can improve adherence, and consequently behavior change.

The term accountability refers to the implicit or explicit expectation that an individual may be called on to justify his or her actions or inactions [72], and being accountable to someone other than oneself enhances motivation to continue with behavioral change. Thus, adherence is an important element to consider in the development of a behavior change intervention. In the model of supportive accountability [11], human support increases adherence through accountability to a virtual coach (ie, a digital person-to-person component) who is seen as trustworthy, benevolent, and having expertise. Adherence will be further enhanced when the relationship with the virtual coach is perceived as reciprocal, clear goals and expectations are defined, and coaches are clear about the accountability process [11]. Moreover, people respond more positively to accountability demands from a coach who is perceived as legitimate [11,73]. Throughout the literature, the use of a virtual health coach has shown positive results in both weight loss [70] and physical activity adherence interventions [74]. Creating social accountability can help individuals self-monitor and follow through on their goals. Furthermore, in the model of supportive accountability [11], there are several human support constructs that are identified (see Figure 1) as integral components to how accountability is cultivated and maintained.

The existence of another human, or social presence [11], can influence accountability and subsequently adherence to behavior change interventions. For example, research suggests that although automated systems that monitor and encourage adherence, such as email reminders, can improve adherence to online interventions, digital person-to-person support enhances adherence to a significantly greater degree [13,29,75]. Expectations of the desired behavior change also play an important part in adherence [11,72]. The more people understand and agree with the fundamental justification for the expected behavior, the greater the compliance. Such expectations need to be not only known but also clear and process, not outcome, focused [11]. Expectations should be monitored; research has shown an important feature of self-monitoring for online weight loss interventions appears to be emailing daily food intake and energy expenditure journals to a weight loss counselor rather than keeping a private record [29,64,76]. Thus, implying further rationale in need for virtual coaches (ie, digital person-to-person) support. It should be noted that the aim of performance monitoring is to provide feedback, to inform that failure to meet goals provides opportunity for self-reflection and growth, and to establish that there are no negative consequences [11]. In addition, it is suggested that supplementing online interventions with feedback and communication components can be effective in creating or generating behavior change [15].

Although all online interventions require participants to act by themselves to some extent, the type and degree of feedback that can be offered by a digital person-to-person component can vary considerably [14], from very little (ie, minimal guidance or supportive feedback mechanism provided) to high (ie, delivery of adequate amounts of tailored feedback). Moreover, immediacy of response is dependent on which communication modality is being employed. Emails and forum postings generally provide delayed feedback, whereas chat room or instant messaging sessions, Skype, and webcam calls provide participants with immediate feedback. Notably, it appears that feedback can be effective whether delivered by the internet [43] or through specific channels such as the use of text messaging [20,48,62]. Use of text messages can allow for immediate feedback on the basis of their response [25], and throughout the literature, the use of text messaging has been found to be a successful behavior change technique [20,48,62,77]. Although differentiating in their degree of direct digital person-to-person contact, feedback channels create an opportunity to foster interpersonal relationships within interactive platforms and create improvements in users’ knowledge, health behavior, clinical outcomes, and social supports [78].

As previously stated, aside from accountability, a virtual coach or online counselor or facilitator aids in creating the feeling of interpersonal connectedness and can provide feedback, which tends to be effective in supportive behavior change [16,75]. Studies have found small to medium effect sizes in internet interventions that incorporate communicative functions such as online advisors [61] and use of an online counselor, compared with no counselor, resulted in greater behavior changes [15]. Similarly, in a Web-based randomized controlled trial that used no counseling, computer-automated feedback (ie, automated tailored messages), or human email counseling (ie, weekly email feedback from a counselor), results indicated that participants who had received computer-automated feedback or human email counseling had better weight loss than those with no counseling [29]. Moreover, in a 3-arm randomized controlled trial, the Facebook Plus group (ie, text messaging, personalized feedback, online support person) had significantly greater weight loss than the Facebook alone and waiting list control groups [25]. Feedback and communication components such as virtual coaches or online facilitators can also help make up an online social support system. Social support networks play an important role in determining health outcomes [79], and as more and more individuals are spending time online, research must examine the role of online social networks and their contribution to health behavior change. In addition, future research must consider the age of the virtual coaches or online facilitators, years of work experience they may have, and their accessibility to digital platforms in the workplace, as these factors play a role in the self-efficacy and utilization of digital platforms in health education organizations [80]. For optimal results, appropriate training for these platforms should be provided to those who will be providing online social support [80].

Increasing social support for a behavior change intervention can be an effective way to enhance desirable outcomes in both traditionally delivered behavioral interventions [79] as well as those delivered online [28,81]. Although utilization and seeking behaviors have been higher in women [82], research suggests that social support may be the most important aspect of online behavior change interventions, as it is the highest predictor of behavior change [48]. Online interventions that do not include some form of social support have lower utilization rates and lack of behavior change [83]. Social support can be encouraged through online social networks. Online social networks, often facilitated through social media platforms and/or a virtual coach/facilitator, have the ability to create high levels of intimacy and immediacy, meaning that support is available despite members’ distance from one another. These characteristics naturally lead to high levels of social support and allow participants to provide each other with social support interactions that are present in face-to-face delivery by adding the possibility of in-the-moment posts and responses [84]. In addition, participants in a study that utilized Facebook [16] were not only successful in supporting one another in a virtual group format, but after the online intervention, the participants reported becoming Facebook “friends.” Evidently, this continued peer support and gained knowledge through digital person-to-person relationships could result in further behavior change.

Online social networks can fill a void in participants, as they increase the feelings of support and connectedness. It has been found that those who reported less baseline social support had lower dropout rates, as the online social network appeared to be filling a gap [49]. Thus, it should be of no surprise that online social networks can be leveraged to foster an online community [85], as many share their personal stories, struggles, or successes [16,24,69,86], fostering a sense of interpersonal connectedness. Moreover, this sense of community can also lead to cyber worlds or communities in which people who used to feel isolated now feel a sense of togetherness [87]. The potential anonymity of online communities is particularly important in cases where health topics may be considered “taboo” or sensitive [21,49,88]. Subsequently, it is important to assess and consider the amount and/or quality of received advice or emotional support provided in online social networks as stress and stigmatization around the health topic can be induced [82]. Online social networks appear to be a predominant component in altering social norms and health behaviors on a large, often times anonymous, and cost-effective scale. Therefore, researchers should examine strategies that will further develop online social support, which can then be used to promote continued adherence and desirable behavior change in online interventions.

## Discussion

### Principal Findings

The growth of the digital environment provides tremendous opportunities to revolutionize health behavior change efforts. Using digital media channels such as Web-based, mobile, or social media in online behavior change interventions can facilitate enhanced communications, research, and education and allows for the generation of multidirectional dialogs [19]. Web-based interventions have used specific behavior change techniques (eg, real-time support, reminders, and sharing platforms) [1] to produce desired behavior change outcomes [6,28,29]. Moreover, the use of mobile technology and social media in delivering health behavior change interventions also produces successful outcomes [38-40,47], and as these platforms continue to rise in popularity, continued efforts should be made to use them in the health research and health advocacy. Overall, digital media channels can be a more cost-effective approach [31] and can have a greater impact on behavior change because of high reach, anonymity, adaptability, and accessibility [1,4] than traditional face-to-face interventions. Furthermore, online technologies have been able to replicate similar results as traditional, in-person interventions [5-8,30]. Thus, online interventions now offer a real alternative, or supplement, to traditional, face-to-face interventions [14]. Although many benefits of using online behavior change interventions are documented, mixed reviews still exist on the delivery of these digital media interventions, whether or not face-to-face interventions are better, and whether in-person components are necessary [36]; thus, future research is justified.

### Conclusions

Overall, face-to-face and online behavior change interventions have their respective advantages and disadvantages (ie, differing degrees of broad reach capability, anonymity, levels of treatment efficacy, and cost) and functions (ie, individual clinical treatment vs public health prevention programs), yet both serve important roles. It is suggested that perhaps the best opportunity for behavior change can be facilitated when there is a combination of face-to-face and online components [1,6,54]. It is the view of the authors that *human support* is the most important component in the effectiveness and adherence of both face-to-face and online behavior change interventions [9-11]. Thus, thoughtfully introducing a digital person-to-person component to replace face-to-face interactions can provide the needed human support while diminishing the barriers of in-person meetings. As human support in face-to-face interventions combines verbal, nonverbal, and contextual cues during in-person communications, a digital person-to-person interaction must implement strategies to overcome these challenges (ie, extended wording, various stylistic procedures for emphasizing text and using emoticons) [14]. Furthermore, using a digital person-to-person component is rooted in social and behavioral theories such as the theory of reasoned action [17] and the social cognitive theory [18] and further justified by the human support constructs of the model of supportive accountability [11]. For example, social comparison, social diffusion and innovation, accountability and adherence, feedback, and perhaps most importantly, social support and connectedness can all be accounted for using digital technology. Therefore, it must be ensured that a digital person-to-person component creates accountability [29,64,76], generates opportunities for tailored feedback [15,20,48,62], and creates social support [84,87]—all key elements in producing successful behavior change. The digital environment is ever changing, and the potential for its use in health behavior change interventions has yet to be fully harnessed [21,24,44,47]. As the popularity of the online world grows and the interest in using the digital environment for health behavior change interventions continues to be embraced, further research into not only the use of online interventions but also the use of a digital person-to-person component must be explored.

## Abbreviations

mHealth: mobile health
